# Who Believes in COVID-19 Conspiracy Theories in Croatia? Prevalence and Predictors of Conspiracy Beliefs

**DOI:** 10.3389/fpsyg.2021.643568

**Published:** 2021-06-18

**Authors:** Mirjana Tonković, Francesca Dumančić, Margareta Jelić, Dinka Čorkalo Biruški

**Affiliations:** Department of Psychology, Faculty of Humanities and Social Sciences, University of Zagreb, Zagreb, Croatia

**Keywords:** COVID-19 conspiracy theories, trust in science and scientists, political powerlessness, authoritarianism, social dominance

## Abstract

The COVID-19 pandemic has given rise to numerous new conspiracy theories related to the virus. This study aimed to investigate a range of individual predictors of beliefs in COVID-19 conspiracy theories that account for sociodemographic characteristics (age, gender, education, economic standard, the importance of religion, and political self-identification), distinctive motivational orientations (social dominance and authoritarianism), relevant social attitudes (sense of political powerlessness and trust in science and scientists), and perceived personal risk (perceived risk for self and family members, the concern of being infected, and the expected influence of pandemic on the economic standard of an individual). Participants were 1,060 adults recruited from the general public of Croatia. The sample was a probabilistic quota sample with gender, age, level of education, size of the dwelling, and region of the country as predetermined quotas. The regression model explained 42.2% of the individual differences in beliefs in COVID-19 conspiracy theories. Trust in science and scientists and political powerlessness were the strongest predictors, whereas fear of being infected had the weakest contribution in explaining the variance of the criterion. Additionally, results revealed that the relation of conventionalism (as a proxy of authoritarianism) with belief in COVID-19 conspiracies was mediated by trust in science and scientists. The relation between social dominance and belief in conspiracies was also partially mediated by trust in science. The results suggest that (re)building trust in science and lowering the sense of political helplessness might help in fighting potentially harmful false beliefs about the pandemic.

## Introduction

A conspiracy theory (CT) may be understood as an alternative explanation of an important social event that is hidden from the public. It almost always implies that a group of powerful individuals secretly manages events solely for their malevolent interests (Bale, 2007). A tendency to believe in conspiracy theories is considered to be a relatively stable mindset or predisposition related to a variety of other cognitive and personality traits and attitudes (Uscinski et al., 2016). Research has shown that tendency to believe in conspiracy theories is related to lower levels of analytic thinking and open-mindedness and higher levels of intuitive thinking (Swami et al., 2014; Pennycook et al., 2015, 2020). It is also related to higher levels of paranoid ideation and schizotypy (Darwin et al., 2011), more pronounced Dark Tetrad traits (Machiavellianism, narcissism, psychopathy, and sadism) (March and Springer, 2019; Bowes et al., 2020), lower agreeableness and conscientiousness (Bowes et al., 2020), as well as lower self-esteem (Swami et al., 2011), and higher individual narcissism (Cichocka et al., 2016).

Beliefs in conspiracy theories are sensitive to social contexts (van Prooijen and Douglas, 2018). Conspiracy theories have been a part of human history for a long time and are more likely to emerge during societal crises driven by a motivation to make sense and establish control and understanding over unpredictable events (van Prooijen and Douglas, 2017). Therefore, it is not surprising that the COVID-19 pandemic gave rise to numerous new conspiracy theories related to the virus, some of which were adopted by many people. At the same time, a growing number of scientific studies are testing accumulated knowledge about predictors, correlates, and consequences of believing in conspiracy theories in the context of the COVID-19 pandemic (for a recent review as shown in van Mulukom et al., 2020). In this study, we will focus on variables predominantly related to social and political factors that may be related to conspiracy ideation (Douglas et al., 2019).

A dual-process motivational approach to ideological attitudes argues that the social and general ideological beliefs are organized along two dimensions: authoritarianism and social dominance (Duckitt, 2001). According to this view, authoritarianism and social dominance express different sets of basic social values or motivational goals. These orientations may have different consequences on how people perceive the world they live in, i.e., on a range of social and political attitudes and behaviors (McFarland and Adelson, 1996; Altemeyer, 1998; Duckitt, 2001). According to Altemeyer (1981, 1996), right-wing authoritarianism (RWA) consists of three attitudinal clusters, authoritarian submission, authoritarian aggression, and conventionalism. Thus, individuals with authoritarian personality are inclined to behave as legitimate authorities tell them, adhere to traditional social norms, and believe that people who do not behave as they are told should be punished. Thus, RWA proved to be an important determinant of prejudice. However, recent studies questioned the notion that RWA is a personality dimension and also showed that authoritarian social attitudes are multidimensionally organized (as shown in Duckitt et al., 2010 for an overview). Thus, newer social psychological theories consider the three authoritarianism subscales as distinct (although related) social attitude dimensions that are expressions of the motivational goal of collective security. On the other hand, according to Sidanius and Pratto (1999), social dominance orientation (SDO) focuses on maintaining existing group-based social hierarchies. More specifically, it recognizes individual differences in the endorsement of group-based hierarchies with some groups at the top and other groups below them, which leads to legitimizing myths that provide justification for these intergroup behaviors and perpetuate hierarchy. It also proved to be an important predictor of prejudice and various political attitudes (McFarland and Adelson, 1996; Sibley et al., 2006). Although these two constructs are weakly correlated, they represent different motivational goals (Duckitt and Sibley, 2009). While social dominance reflects the beliefs of an individual about the extent to which the world is a competitive jungle, authoritarianism reflects beliefs of the world as a dangerous place. Thus, both could be triggered by the COVID-19 pandemic as a threat to either social hierarchy or security (Duckitt and Fisher, 2003; Huang and Liu, 2005).

Previous studies of conspiracy ideation examined the relative role of ideological orientations or beliefs, authoritarianism and social dominance, in explaining the variance of beliefs in conspiracy theories. The relationship between ideological attitudes and conspiracy ideation was confirmed, people with higher levels of authoritarianism (Abalakina-Paap et al., 1999), RWA, and SDO (Swami, 2012; Bruder et al., 2013; Imhoff and Bruder, 2014) were more likely to believe in conspiracy theories. To our knowledge, until recently, there is only one study on COVID-19 conspiracy theories that included measures of both authoritarianism and social dominance. Results indicated that both variables were positively correlated with belief in COVID-19 conspiracy theories (Lobato et al., 2020).

Abalakina-Paap et al. (1999) proposed five types of reasons of why people believe in conspiracy theories: alienation, powerlessness, simplification of the complex world, explanation of their problems, and providing an opportunity for their hostility. Since conspiracy theories typically imply that a group of powerful people stands behind important events and controls the lives of others in secret, individuals who are distrustful of others and authorities may be prone to explanations offered by conspiracy theories. A feeling of alienation is often accompanied by a feeling of powerlessness, and conspiracy theories provide an expalantion for individual hardship. Similarly, people who feel they have a disadvantaged position in society can adhere to conspiracy theories as an explanation. Indeed, believing in conspiracies was found to be related to higher levels of anomie and powerlessness (Abalakina-Paap et al., 1999; Bruder et al., 2013) and anomie and lower interpersonal trust (Brotherton et al., 2013). Political powerlessness mediated the relationship between conspiracy beliefs and behavioral intentions in the case of vaccination and climate preserving behaviors (Jolley and Douglas, 2014a,b). The relationship between powerlessness and believing in conspiracies was confirmed in the context of COVID-19 conspiracies (Biddlestone et al., 2020).

Since the official explanation of events that conspiracy theories dominantly focus on (such as vaccination, landing on the moon, chemtrails, etc.) is almost always scientific, belief in such theories should be closely associated with mistrust and negative attitudes toward science. Although this connection might seem straightforward (Hartman et al., 2017), most studies focused on the rejection of a specific scientific field as a potential adverse effect of spreading conspiracy theories. For example, it has been shown that conspiratorial thinking is related to the rejection of climate science (Lewandowsky et al., 2013a,b; van der Linden, 2015). On the other hand, only a few studies examined general trust in science and scientists as a predictor of acceptance of conspiracy theories. In the context of belief in COVID-19 conspiracy theories, research showed that science skepticism was strongly associated with endorsement and willingness to spread COVID-19 conspiracies (Lobato et al., 2020) and that trust in scientists was negatively related to believing in COVID-19 conspiracies (Constantinou et al., 2020). Furthermore, trust in science had a mediating role in the relationship between conspiracy ideation and willingness to accept prevention guidelines (Plohl and Musil, 2020).

Although trust in science may be highly influenced by contextual factors, such as the current epidemic, some studies indicated that more stable characteristics may also have an influence, e.g., Walter et al. (2001) report SDO to be correlated with feelings of suspicion whereas RWA is correlated with “irrational” beliefs. These feelings and beliefs directly contravene science and trust in it. Hence, it is expected that RWA and SDO might predict conspiracy belief by reducing trust in science. Although, to our knowledge, there are no studies that investigated the role RWA and SDO play in trust in science, we build upon a research study that looked at the relationship between RWA, SDO, and trust in various public institutions in a longitudinal perspective (Castillo et al., 2011). In this study, RWA and SDO showed significant associations with trust in all public institutions (some correlations were positive and some negative), and the longitudinal nature of this study also revealed that some correlations reversed their effects in time due to modifications in the characteristics of the governmental institutions (e.g., elections and change in the political structure). In addition, RWA was more sensitive to the situational changes in threat than SDO (Doty et al., 1991; Duckitt and Fisher, 2003). Although the trust in science in Croatian society is generally high (Prpi, 2011; Šuljok, 2020), in the case of pandemics, trust in science usually declines and is replaced with public skepticism with the passage of time and increasing economic consequences (Bucchi and Saracino, 2020). Thus, we expect that the threat brought by COVID-19 increases perceived competition and danger among those high on RWA and SDO (resulting in reduced trust in science) and that trust in science might have a mediating role in the relationship between RWA and SDO and conspiracy beliefs.

Unlike some other events that conspiracy theories are focused on, the COVID-19 pandemic offers a rare opportunity to examine the role of individual experiences related to the pandemic, such as perceived personal risk, on accepting unfounded beliefs about the virus. The COVID-19 pandemic poses a global threat, both to the economy and health, and comes with a great many unknowns. This makes it a perfect setting for the rise of anxiety levels and the creation of new conspiracy theories. Research has confirmed a positive correlation between anxiety about COVID-19 and the belief that the disease is part of a conspiracy (Sallam et al., 2020) and between personal uncertainty and conspiratorial thinking (Miller, 2020). COVID-19 conspiracy beliefs were positively correlated with COVID-19 risk perception and anxiety about the virus, and the effect of risk perception on COVID-19-specific conspiracy beliefs was fully mediated by anxiety (Šrol et al., 2021). Risk perception was defined not as an individual, but as a general, risk in terms of perceived infectiousness, severity, and dangerousness of COVID-19. However, research has not yet examined the potential contribution of the perceived personal economic risk of the pandemic on belief in conspiracy theories. Nevertheless, personal risk, in terms of risk to the health or economic status of an individual, might prove to be a positive predictor and explain an additional portion of the variance in conspiracy beliefs over and above more stable social factors described earlier.

Believing in conspiracy theories about COVID-19 has numerous adverse consequences from reduced safeguarding behavior and adherence to protective guidelines to pseudoscientific health practices (van Mulukom et al., 2020). Identifying vulnerable groups who are prone to believing in conspiracy theories is, therefore, of high importance. Finally, the majority of studies on COVID-19 conspiracy theories used convenience samples with a disproportionately higher number of women, young, and more educated participants than in the general population.

This study aimed to simultaneously investigate a range of individual predictors of beliefs in COVID-19 conspiracy theories in the Croatian general population. Predictors can be arranged into four distinct groups: sociodemographic characteristics (age, gender, education, economic standard, the importance of religion, and political self-identification), distinctive motivational orientations (social dominance and authoritarianism), relevant social attitudes (sense of political powerlessness and trust in science and scientists), and perceived personal risk (perceived personal and/or family member vulnerability, the concern of being infected, and the expected influence of pandemic on the economic standard of an individual). We hypothesized that each of these individual attributes significantly contributes to explaining individual differences in belief in COVID-19 conspiracy theories. We expected the importance of religion, social dominance, authoritarianism, powerlessness, perceived risk for self and family members, a concern of being infected, and the influence of pandemic on the economic standard to be positive predictors of beliefs in COVID-19 conspiracy theories, while the level of education, political self-identification, trust in science and scientists were expected to be negative predictors of belief in COVID-19 conspiracy theories. Furthermore, we expected that each group of predictors would have an incremental contribution toward explaining individual differences in belief in COVID-19 conspiracy theories above and beyond preceding predictors. We also expected trust in science and scientists to mediate the relationship between social dominance and belief in conspiracies and the relationship between authoritarianism and belief in conspiracies. The structure of our sample enables us to explore the level of acceptance of various conspiracy theories about COVID-19 in Croatia.

## Materials and Methods

### Participants

Data collection were done as a part of a larger project using an online panel of respondents who were compensated for their time. Participants were compensated for their time and effort by the system of rewarding points developed by the agency that collected the data. The sample was a national probabilistic quota sample that was two-way stratified: by region and by size of the dwelling. The structure of the total sample corresponded to the structure of the targeted population according to the valid census, including the distribution of age, gender, and, with corrections, education. Participants comprised 1,060 adults recruited from the general population of Croatia. The mean age was *M* = 44 years (*SD* = 15.8; min = 18; max = 74). About 53.1% of participants were women. Regarding education, there were 16.3% of participants with unfinished or finished primary education, 58.9% finished middle education, and 24.8% were highly educated.

### Measures

This research is part of a larger project aimed at targeting determinants of the post-corona social recovery of the Croatian society (Čorkalo Biruški et al., 2020), but we will describe only measures relevant for this article. Participants responded to several other measures related to the main goals of the project.

#### Demographics

Participants were asked about their age, gender, education level, and estimated economic standard compared to other households in Croatia (ranging from 1 = *significantly below average* to 5 = *significantly above average*), the importance of religion (ranging from 1 = *not important at all* to 5 = *extremely important*), and political self-identification (from left to right with an option to declare oneself as having no political identification).

#### Right-Wing Authoritarianism

Authoritarianism was measured with a very short authoritarianism scale (Bizumic and Duckitt, 2018). This is a 6-item scale with two items representing each of the three content subdimensions: conservatism or authoritarian submission, traditionalism or conventionalism, and authoritarianism or authoritarian aggression. Item example from conventionalism subdimension: *God's laws about abortion, pornography, and marriage must be strictly followed before it is too late*. Responses were given on a 5-point Likert scale (1 = *extremely disagree*; 5 = *extremely agree*). Reliability analysis for the whole scale resulted in Cronbach's α of only 0.48 with very low inter-item correlations except for the correlation of two items representing conventionalism (*r* = 0.54; *p* < 0.01). Therefore, we decided not to use the whole scale in further analyses but only the subscale of conventionalism/traditionalism. Cronbach's α for this subscale was 0.69. We use it further as representing a proxy measure of authoritarianism.

#### Social Dominance Orientation

Social dominance orientation was measured with the 5-item Group Dominance subscale (adapted from Todosijević, 2013) of the SDO Scale (Pratto et al., 1994). Participants indicated their agreement with statements such as *In getting what your group wants, it is sometimes necessary to use force against other groups* on a 5-point Likert scale (1 = *extremely disagree*; 5 = *extremely agree*). Confirmatory factor analysis (CFA) confirmed good fit for the expected one-factor model (χ^2^ = 112; *df* = 5; *p* < 0.001 *RMSEA* = 0.14; *CFI* = 0.93; *TLI* = 0.87) and the obtained Cronbach's α was satisfactory (α = 0.81).

#### Political Powerlessness

Political powerlessness was measured with a 10-item scale by Neal and Groat (1974). The scale was conceptualized as a low expectancy for control over the outcomes of events and is limited to the political and economic aspects (Neal and Groat, 1974). Participants indicated their agreement with statements such as *It is only wishful thinking to believe that one can influence what happens in society at large* on a 5-point Likert scale (1 = *extremely disagree*; 5 = *extremely agree*). Exploratory factor analysis (EFA) suggested a three-factor structure, with six items loading on the first factor, two items loading on the second, and two items loading on the third factor (χ^2^ = 97.4; *df* = 18; *p* < 0.001 *RMSEA* = 0.7; *TLI* = 0.91). Items loading on the second and the third factor were the ones that required reverse scoring^1^. Since we could not identify any meaningful difference in the content of those items and items loading on the first factor, we decided to keep only items that had sufficient loadings on the first factor. Cronbach's α for the remaining six items was 0.73.

#### Trust in Science and Scientists

Trust in science and scientists was measured with the Trust in Science and Scientists Inventory (Nadelson et al., 2014), which originally contains 21 items but was shortened for this study to 12 items, based on previously collected data (Peterlin, 2019). Participants indicated their agreement with statements such as *I trust scientists can find solutions to our major technological problems* on a 5-point Likert scale (1 = *extremely disagree*; 5 = *extremely agree*). EFA resulted with two correlated factors and CFA confirmed good fit for the model with one higher-order factor (χ^2^ = 200.6; *df* = 52; *p* < 0.001; *RMSEA* = 0.05; *CFI* = 0.97; TLI = 0.97). Cronbach's α was satisfactory (α = 0.88).

#### Perceived Personal Risk

The perceived personal risk was measured with four items. Participants were asked whether they belong to the group at a higher risk of getting infected with COVID-19, whether someone from their family belongs to the group at a higher risk of getting infected with COVID-19, how concerned they are about getting COVID-19 in the future (from 0 = *not at all concerned* to 10 = *extremely concerned*) and to rate the expected influence of pandemic on their economic standard in comparison to other citizens of Croatia (from 1 = *much less than on others* to 5 = *much more than on others*).

#### Beliefs in COVID-19 Conspiracy Theories

Belief in COVID-19 conspiracy theories was measured with an *ad-hoc* scale constructed for the purpose of this study and consisted of 10 items. Participants were asked to indicate their agreement with statements describing common and popular conspiracy theories about COVID-19 such as *The coronavirus pandemic is the result of a large pharmaceutical companies agreement to make money on vaccines*. or *The coronavirus spreads faster in the presence of 5G networks*. Responses were given on a 5-point Likert scale (1 = *extremely disagree*; 5 = *extremely agree*). EFA resulted with two correlated factors and CFA confirmed good fit for the model with one higher-order factor (χ^2^ = 327.3; *df* = 33; *p* < 0.001; *RMSEA* = 0.09; *CFI* = 0.96; *TLI* = 0.94). Cronbach's α was high (α = 0.92).

### Procedure

Data were collected from August 24, 2020 to September 7, 2020 with the help of a well-established public opinion research agency using the computer-assisted web interviewing (CAWI) method. The measures of interest were presented in four blocks: (a) demographics, (b) block containing RWA and SDO scales, (c) block containing trust in science and scientists and political powerlessness scales, and (d) block containing questions about perceived personal risk and beliefs in COVID-19 conspiracy theories scale. Participants answered the demographic questions first, while the order of the three remaining blocks was counterbalanced. Furthermore, the order of the scales in each block was randomized except in block (d). It took the participants ~35 min to complete the questionnaires. At the end of the questionnaire, participants were provided with information about resources where they could ask for psychological support and/or help if they felt they needed it.

## Results

Before investigating predictors of belief in COVID-19 conspiracy theories, we were interested in the endorsement rate of such theories in our sample. As can be seen in Table 1 that reports the question wordings and percent of respondents who agree or strongly agree with each CT, the majority of participants agreed with the conspiracy about the real number of infected people being hidden, followed by the theory about laboratory origin of the virus that almost one-half of the participants agreed with. Another four theories were endorsed by more than a third of the participants.

**Table 1:** COVID-19 conspiracy theories endorsement rates.

**Question wording**	**% of agree or strongly agree responses**
The true number of people infected with coronavirus is hidden from the public.	58.58
The coronavirus did not originate from animals but was created by scientists in the laboratory.	45.09
The coronavirus was released with the aim of destroying some of the world's economies.	38.68
World governments are using this pandemic to abolish civil liberties.	36.32
The coronavirus is as serious as the common flu, if not less so.	35.28
The coronavirus pandemic is the result of a large pharmaceutical companies' agreement to make money on vaccines.	34.34
The coronavirus vaccine already exists, but it is currently being kept secret from us.	25.75
The story about the coronavirus was placed in order to implant a chip with a “developed vaccine.”	17.83
The Bill and Melinda Gates Foundation is responsible for the creation and spread of the coronavirus.	15.47
The coronavirus spreads faster in the presence of 5G networks.	10.38

Results showed that the general level of belief in COVID-19 conspiracy theories in our sample was a little below the middle point of the scale (*M* = 2.89; *SD* = 0.93), with 23% of participants having an average score >3.5, indicating that they strongly agree or agree with conspiracy theories. In comparison to the general sample, participants who (strongly) agree with conspiracy theories have lower estimated economic standards, have lower level of education, showed more importance to religion, and are more likely not to self-identify themselves politically (Tables 2, 3).

**Table 2:** Some sociodemographic characteristics of participants in a general sample compared to the same characteristics among participants who (strongly) agree with COVID-19 conspiracy theories.

	**Frequency in a general sample**	**% of participants who agree or strongly agree with the CTs**
**Gender**		
Female	563	22.74%
Male	497	23.34%
**Education**		
Less than elementary school	10	20.00%
Elementary school	163	29.45%
High school	624	23.72%
University	253	17.79%
PhD	10	10.00%
**Political self-identification**		
Not politically self-identified	492	27.85%
Politically self-identified	568	18.84%
*N*	1,060	23.00%

**Table 3:** Correlation matrix and descriptive statistics.

	**General sample**		**Participants who agree or strongly agree with the CTs**															
	***M***	***SD***	***M***	***SD***	**1**	**2**	**3**	**4**	**5**	**6**	**7**	**8**	**9**	**10**	**11**	**12**	**13**	**14**
1. Belief in COVID-19 conspiracy theories	2.89	0.93	4.14	0.41	—													
2. Age	44.01	15.76	41.74	14.30	−0.06	—												
3. Gender					0.05	−0.01	—											
4. Education					−0.18^**^	−0.11^**^	−0.05	—										
5. Economic standard	2.9	0.75	2.82	0.83	−0.10^**^	−0.13^**^	−0.04	0.24^**^	—									
6. The importance of religion	3.14	1.35	3.43	1.33	0.22^**^	0.06^*^	0.07^*^	−0.12^**^	−0.06^*^	—								
7. Political self–identification					−0.23^**^	0.17^**^	−0.14^**^	0.14^**^	0.10^**^	−0.06	—							
8. Social dominance	2.28	0.86	2.52	0.98	0.25^**^	−0.03	−0.14^**^	−0.09^**^	<−0.01	0.09^**^	<0.01	—						
9. Authoritarianism	2.14	0.99	2.29	1.05	0.20^**^	0.11^**^	0.03	−0.14^**^	−0.11^**^	0.52^**^	−0.04	0.19^**^	—					
10. Political powerlessness	3.42	0.69	3.72	0.74	0.32^**^	0.10^**^	0.09^**^	−0.14^**^	−0.15^**^	0.07^*^	−0.10^**^	0.14^**^	−0.01	—				
11. Trust in science and scientists	3.24	0.61	2.78	0.54	−0.60^**^	0.02	−0.04	0.18^**^	0.11^**^	−0.19^**^	0.23^**^	−0.26^**^	−0.27^**^	−0.30^**^	—			
12. Perceived risk for self	0.26	0.44	0.21	0.41	−0.05	0.42^**^	<−0.01	−0.11^**^	−0.13^**^	0.04	0.13^**^	−0.03	0.09^**^	0.04	0.04	—		
13. Perceived risk for family members	0.51	0.50	0.49	0.50	−0.04	−0.18^**^	0.09^**^	0.05	0.08^*^	−0.06	<−0.01	<0.01	−0.07^*^	−0.02	0.05	−0.17^**^	—	
14. Concern of being infected	4.78	3.39	4.32	3.76	−0.06	0.18^**^	0.09^**^	−0.10^**^	−0.08^**^	0.11^**^	0.06	0.04	0.13^**^	0.10^**^	0.01	0.24^**^	0.10^**^	—
15. Expected influence of pandemic on one's economic standard	3.06	0.67	2,95	0.80	0.10^**^	0.06	0.04	−0.03	−0.20^**^	0.05	−0.02	−0.10^**^	0.04	0.06	−0.06^*^	0.03	−0.02	0.03

* *p <0.05*;

** *p < 0.01*.

As already mentioned, we hypothesized that sociodemographic characteristics, distinctive motivational orientations, relevant social attitudes, and perceived personal risk significantly contribute to explaining individual differences in belief in COVID-19 conspiracy theories. Before any analysis, a correlation matrix was checked (Table 3). Intercorrelations between predictor variables were not high (the highest correlation was obtained between the importance of religion and the conventionality subscale of authoritarianism; *r* = 0.52), but the majority of predictor variables were correlated with the criterion variable. Importance of religion, social dominance, conventionalism, political powerlessness, and the expected influence of pandemic on the economic standard of an individual were positively correlated with believing in COVID-19 conspiracies, while the level of education, economic standard, political self-identification, and trust in science and scientists were negatively correlated with the criterion variable.

Since we were interested both in the contribution of each predictor to explaining individual differences in belief in COVID-19 conspiracy theories and to explaining the amount of variance of criterion variable accounted for by each group of predictors after controlling predictors from earlier groups, a four-step multiple regression was conducted with belief in COVID-19 conspiracy theories as to the criterion variable. Sociodemographic variables (age, gender, education, economic standard, the importance of religion, and political self-identification) were entered in step one, motivational orientations (social dominance and authoritarianism) were entered in step two, social attitudes (sense of political powerlessness and trust in science and scientists) were entered in step three and perceived personal risk (perceived risk for self and family members, a concern of being infected, and the expected influence of pandemic on the economic standard of an individual) were entered in step four. Variables were entered in order of their stability, so the variables less susceptible to change (i.e., sociodemographic variables and more enduring motivational orientations) were entered before contextual variables. Regression statistics are shown in Table 4.

**Table 4:** Summary of hierarchical regression analysis for variables predicting the belief in COVID-19 conspiracy theories.

	**Step 1**			**Step 2**			**Step 3**			**Step 4**		
**Variable**	***B***	***SE***	**β**	***B***	***SE***	**β**	***B***	***SE***	**β**	***B***	***SE***	**β**
Age	<−0.01	<0.01	−0.05	<−0.01	<0.01	−0.05	<−0.01	<0.01	−0.06^*^	<−0.01	<0.01	−0.05
Gender	−0.01	0.06	−0.01	0.06	0.05	0.03	0.01	0.05	0.01	0.02	0.05	0.01
Education	−0.17	0.04	−0.12^**^	−0.13	0.04	−0.10^**^	−0.06	0.04	−0.04	−0.07	0.04	−0.05
Economic standard	−0.06	0.04	−0.05	−0.06	0.04	−0.05	−0.01	0.03	−0.01	0.01	0.03	0.01
The importance of religion	0.13	0.02	0.19^**^	0.10	0.02	0.14^**^	0.07	0.02	0.10^**^	0.07	0.02	0.10^**^
Political self–identification	−0.35	0.06	−0.19^**^	−0.36	0.06	−0.19^**^	−0.16	0.05	−0.09^**^	−0.15	0.05	−0.08^**^
Social dominance				0.24	0.03	0.22^**^	0.10	0.03	0.09^**^	0.11	0.03	0.11^**^
Authoritarianism				0.06	0.03	0.07^*^	<0.01	0.03	<0.01	0.01	0.03	0.01
Political powerlessness							0.20	0.04	0.15^**^	0.21	0.04	0.15^**^
Trust in science and scientists							−0.73	0.04	−0.48^**^	−0.71	0.04	−0.47^**^
Perceived risk for self										<0.01	0.06	<−0.01
Perceived risk for family members										−0.01	0.05	−0.01
Concern of being infected										−0.02	0.01	−0.08^**^
Expected influence of pandemic on one's economic standard										0.10	0.03	0.07^**^
*R^2^*	0.115			0.170			0.412			0.422		
Δ*R^2^*	0.115^**^			0.055^**^			0.242^**^			0.010^**^		

* *p < 0.05*;

** *p < 0.01*.

The hierarchical multiple regression revealed that sociodemographic variables entered in step one accounted for 11.5% of the variation in belief in COVID-19 conspiracy theories. Participants with lower education, those to whom religion is more important, and those who are politically non-identified, were more likely to believe in conspiracy theories. Motivational orientations entered in step two explained an additional 5.5% of variation in belief in COVID-19 conspiracy theories and this change was significant [*F*_(2, 1,042)_ = 42.54; *p* < 0.001]. Participants with higher social dominance and authoritarianism scores were more likely to believe in conspiracies. Adding social attitudes to the regression model in step three explained an additional 24.2% of the variance in a criterion variable. This change was also significant [*F*_(2, 1,040)_ = 214.31; *p* < 0.001]. While a sense of political powerlessness was a positive predictor of belief in COVID-19 conspiracy theories, trust in science and scientists was a negative predictor. In this step, level of education and authoritarianism were not significant predictors of belief in COVID-19 conspiracy theories any more. Finally, the addition of perceived personal risk to the regression model in step four explained another 1% of the variance in a criterion variable [*F*_(4, 1,036)_ = 4.51; *p* < 0.01]. Only the concern of being infected and the expected influence of pandemic on the economic standard of an individual were significant predictors of a criterion variable. Those participants that are more concerned about being infected are less prone to believing in conspiracy theories, while those that expect pandemic to have a greater influence on their economic standard are more prone to believing in conspiracy theories. Generally, the most important predictor was trust in science and scientists. Taken together, all predictors accounted for 42.2% of the variance of belief in conspiracy theories.

Finally, we expected trust in science and scientists to mediate the relationship between authoritarianism and belief in COVID-19 conspiracy theories and the relationship between social dominance andbelief in conspiracies. Therefore, two mediation analyses were conducted.

**Figure 1:**
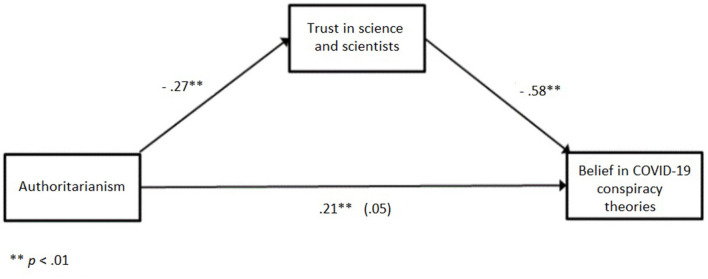
Standardized regression coefficients for the relationship between authoritarianism and belief in COVID-19 conspiracy theories as mediated by trust in science and scientists.

The relationship between authoritarianism and belief in COVID-19 conspiracy theories was fully mediated by trust in science and scientists. As Figure 1 illustrates, the standardized coefficient between authoritarianism and trust in science and scientists was statistically significant (*a* = −0.27: *p* < 0.01), as was the standardized coefficient between the trust in science and scientists and belief in COVID-19 conspiracy theories (*b* = −0.58: *p* < 0.01). The standardized direct effect was not significant (*c'* = 0.05: *p* > 0.05). The standardized indirect effect, however, was significant (*ab* = 0.16: *p* < 0.01). We tested the significance of this indirect effect using bootstrapping procedures. Unstandardized indirect effects were computed for each of 5,000 bootstrapped samples, and the 95% confidence interval was computed by determining the indirect effects at the 2.5th and 97.5th percentiles. The bootstrapped unstandardized indirect effect was 0.15, and the 95% confidence interval ranged from 0.11 to 0.18. Thus, the indirect effect was statistically significant. Participants with higher scores on authoritarianism are less likely to trust science and scientists, thus they are more likely to believe in COVID-19 conspiracy theories.

**Figure 2:**
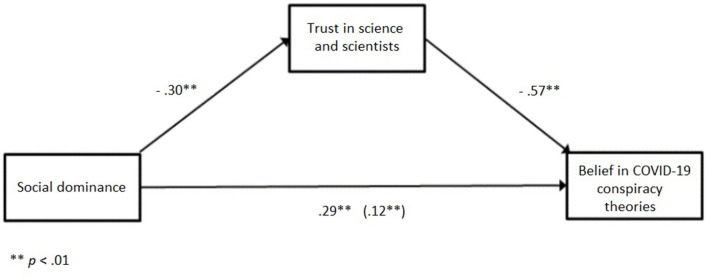
Standardized regression coefficients for the relationship between social dominance and belief in COVID-19 conspiracy theories as mediated by trust in science and scientists.

The relationship between social dominance and belief in COVID-19 conspiracy theories was partially mediated by trust in science and scientists. As can be seen in Figure 2, the standardized coefficient between social dominance and trust in science and scientists was statistically significant (*a* = −0.30: *p* < 0.01), as was the standardized coefficient between trust in science and scientists and belief in COVID-19 conspiracy theories (*b* = −0.57: *p* < 0.01). The standardized direct effect was also significant (*c'* = 0.12: *p* < 0.01). The standardized indirect effect was also significant (*ab* = 0.17: *p* < 0.01). Again, the significance of indirect effect was tested using bootstrapping procedures, following the previously described steps. The bootstrapped unstandardized indirect effect was 0.16, and the 95% confidence interval ranged from 0.12 to 0.20. Thus, the indirect effect was statistically significant. Participants with scores higher on social dominance are less likely to trust science and scientists, so they are more likely to believe in COVID-19 conspiracy theories.

To sumamrize, the relationship between authoritarianism and the belief in COVID-19 conspiracies was fully mediated by trust in science and scientists, while the relationship between social dominance and belief in conspiracies was partially mediated by trust in science.

## Discussion

The present study aimed to investigate the predictors of believing in COVID-19 conspiracies in the general population of Croatia and to examine the potential influence of experiences with the disease in explaining the additional variance of conspiracist beliefs.

Almost a quarter of the participants had an average score, indicating that they agree or strongly agree with COVID-19 conspiracy theories. The results showed that believing in COVID-19 conspiracies was associated with lower education, lower economic standard, higher importance of religion, and declaring no political self-identification. As expected, we found no differences in believing in conspiracy theories related to COVID-19 based on gender and no relationship with the age of the participants. Results about age and gender differences in believing in COVID-19 conspiracy theories in previous research are mixed with no clear pattern of gender or age differences. Some studies found no gender differences (Earnshaw et al., 2020; Freeman et al., 2020), some found that women are more likely to believe in COVID-19 conspiracy theories (Alper et al., 2020; Erceg et al., 2020; Patsali et al., 2020), while some found that men are more likely to endorse COVID-19 conspiracy theories (Cassese et al., 2020). Similarly, correlation with age was found in studies by Freeman et al. (2020), Constantinou et al. (2020), Earnshaw et al. (2020), although the correlation differed in sample size.

Regarding education, income, and religiosity, previous research demonstrated relationships in line with lower-income and education levels (Constantinou et al., 2020; Hornik et al., 2021) and a higher level of religiosity (Alper et al., 2020), which were related to a higher endorsement of COVID-19 conspiracy theories.

Since 46.4% of the participants declared not having a political self-identification, we additionally calculated the correlation between political identification and believing in COVID-19 conspiracies on a subsample of participants who did provide an answer to the question about political orientation on a scale from extremely left/liberal to extremely right/conservative. The analysis showed a positive correlation (*r* = 0.14; *p* < 0.001) in line with a previously obtained positive relationship of conservatism and right-wing ideology with an endorsement of pandemic unrelated conspiracies (Swami, 2012; Pasek et al., 2015; Douglas et al., 2016) and pandemic related conspiracies (Alper et al., 2020; Calvillo et al., 2020; Miller, 2020; Romer and Jamieson, 2020; Uscinski et al., 2020; Farias and Pilati, 2021).

Believing in COVID-19 conspiracy theories was related to a higher result on the SDO scale, higher authoritarianism, higher powerlessness, and lower trust in science and scientists. While other correlations were low or at best moderate in size, correlation with trust in science and scientists is the highest obtained in the study. Trust in science and scientists should be differentiated from science curiosity, science literacy, or scientific reasoning. While the latter represent the ability or willingness to comprehend scientific knowledge, trust in science is more of an attitude toward science and scientists as an authority. Therefore, the observed correlation between trust in science and scientists and believing in conspiracies is expected and fits well in the conspiracy mentality of people who are prone to believe in conspiracy theories. Furthermore, many COVID-19 conspiracies imply that scientists and science are to be blamed for the pandemic (e.g., COVID-19 originated from the science laboratory, COVID-19 vaccine exists but it is kept secret, the real number of infected people is hidden, etc.).

The perceived personal risk was not related to the belief in conspiracies, except for the expected influence of pandemic on the standard of an individual in comparison to others. Participants who expect a higher personal impact from the pandemic believe more in conspiracies.

The regression model explained 42.2% of the individual differences in beliefs in COVID-19 conspiracy theories. Trust in science and scientists and political powerlessness were the strongest predictors, whereas concern of being infected had the weakest contribution in explaining the variance of the criterion. The importance of religion, social dominance, powerlessness, and expected greater influence of pandemic on the standard of an individual were positive predictors, while political self-identification, trust in science and scientists, and concern about being infected were negative predictors, of believing in COVID-19 conspiracies.

Additionally, results confirmed that the relationship between authoritarianism and belief in COVID-19 conspiracy theories was mediated by trust in science and scientists. The relationship between social dominance and belief in conspiracies was also partially mediated by trust in science. This finding is in line with our expectations and shows that in times of prolonged threat, such as the COVID-19 pandemic, trust in science is declining and being replaced with public skepticism as people face the economic consequences (as suggested by Bucchi and Saracino, 2020). The sense of threat caused by COVID-19 might especially have affected those high on RWA and SDO who perceive those in charge as less trustful and not responding properly to the outside danger. As scientists now hold the key to “normality,” this might have resulted in reduced trust in science, which in turn leads to an increase in endorsing conspiracy beliefs. As RWA proved to be more sensitive to contextual changes than SDO (Doty et al., 1991; Duckitt and Fisher, 2003; Castillo et al., 2011), it comes as no surprise that mediation is stronger in this case.

As previously mentioned, believing in conspiracies is related to numerous adverse behaviors which are particularly undesirable in times of pandemic, such as unwillingness to adhere to protective guidelines (Farias and Pilati, 2021; Karić and Mededović, 2021; Soveri et al., 2021). In line with that, compliance with protective measures is related to a similar set of variables. For example, civic attitudes (Roma et al., 2020) and trust in science and scientists (Dohle et al., 2020; Hromatko et al., 2021) are found to be positively related to adherence to COVID-19 protective measures. However, while some previous research, consistent with this study, show that education is negatively related to beliefs in conspiracies (Hornik et al., 2021), some other show that education is not a significant predictor (Hromatko et al., 2021; Karić and Mededović, 2021) or is even negatively related to compliance to protective measures (Roma et al., 2020). These results suggest that the relationship of education with belief in conspiracies and adherence to protective measures is not straightforward. It is for further research to unfold this relationship in more detail.

### Strengths and Limitations of the Study

The present study has several strengths, including a large national probabilistic sample and the fact that data were collected after the first wave of the pandemic was over in Croatia, but conspiracy theories were on the rise. Future studies should monitor the trajectory of such beliefs, both regarding their content and the extent to which people believe in them, as well as determinants of such beliefs.

Nevertheless, an important limitation of the study is the online panel sample that could have introduced a selection bias, as only those who own a device and are internet users were able to participate as suggested by the low proportion of the uneducated participants in our sample. Nevertheless, as of the beginning of 2019, almost 80% of Croatians aged between 16 and 74 years used the internet (Eurostat, 2020). That being said, it should also be noted that conspiracy theories are dominantly spreading through the internet (Bessi et al., 2015), which additionally justifies using an online panel. Second, this survey was based on self-report instruments, some of which were first used in this study. Even though some were successfully used in previous studies, e.g., the authoritarianism scale (Bizumic and Duckitt, 2018), the reliability of specific subscales proved to be too low, and hence, we used only the conventionalism subscale as the proxy for authoritarianism as it proved to be the most reliable. Moreover, even in the original study, the internal reliability for a 2-item Conventionalism subscale was far higher (i.e., >0.70) than for two other subscales (Bizumic and Duckitt, 2018). There is a plausible expectation that, in times of uncertainty and collective crises, one way of coping with anxiety and distress for some people may be to turn to more dogmatic, authoritarian views and intolerance toward those who oppose “law and order” (e.g., Merolla et al., 2011). Since conventionalism (i.e., traditionalism) precisely reflects a tendency to keep things as they are and to insist on preserving the status quo, which are values at the core of authoritarianism, we used the conventionalism scale as a proxy of authoritarianism. Although we acknowledge a tripartite nature of the authoritarian orientations we have departed from, we also emphasize that conventionalism in itself can increase the belief in conspiracy theories. There is no doubt that the COVID-19 pandemic has caused a threat to our everyday lives. Hence, believing in conspiracy theories may serve as a coping mechanism in dealing with a shaken social order. When a threat arises, those who insist strongly on traditional values and defend their current way of life find it difficult to cope, so a likely coping mechanism may seem to be to believe that “someone” is trying to disturb the order of things and/or destroy traditional values as a part of a “conspiracy” against traditional morality and social values. This finding is also in line with newer socio-psychological theories of RWA that imply that the three dimensions are distinct (Feldman, 2003; Kreindler, 2005; Jugert and Duckitt, 2009; Duckitt et al., 2010). Some studies already showed that they differentially predict interpersonal behavior, social policy support, and political party support (Duckitt et al., 2010), and the Conventionalism scale proved to be different from the two other dimensions in some previous studies as well (Feldman, 2003; Stenner, 2005). Therefore, our result might complement these findings and imply that conceptualizing authoritarianism as a set of three related, but distinct, ideological attitude dimensions may be more applicable for explaining complex socio-political phenomena than the unidimensional model. Nevertheless, it is theoretically sound to expect that two other dimensions of the RWA syndrome, i.e., authoritarian submission and authoritarian aggression, may also be predictive for believing in CT. These topics are for future studies to explore these relationships more thoroughly.

Conspiracy theories are an ever-existing part of society, but possible ways of fighting against them are still not clear. Results of the study revealed a strong relationship between trust in science and scientists and belief in conspiracy theories and a sense of political powerlessness and belief in conspiracies. Although the correlational nature of this study prevents us from making any causal claims, results suggest that (re)building trust in science and scientists and lowering the sense of political helplessness might help to fight any potentially harmful false beliefs about the pandemic. This path might be especially important for people with high scores in authoritarianism and those with high scores in social dominance. Finally, this study highlights that a personal experience is not highly important for succumbing to irrational beliefs, as proven with a weak contribution of perceived personal risk in explaining CT beliefs.

## Data Availability Statement

The datasets presented in this study can be found in online repositories. The names of the repository/repositories and accession number(s) can be found at: The dataset generated for this study can be found in the CROSSDA (Croatian Social Science Data Archive) https://doi.org/10.23669/CLYQHG.

## Ethics Statement

The studies involving human participants were reviewed and approved by Ethics Committee of the Department of Psychology, Faculty of Humanities and Social Sciences, University of Zagreb. The patients/participants provided their written informed consent to participate in this study.

## Author Contributions

MT, FD, MJ, and DČ contributed to conception and design of the study. FD organized the database. MT and FD performed the statistical analysis. MT wrote the first draft of the manuscript. MJ, FD, and DČ wrote sections of the manuscript. All authors contributed to manuscript revision, read, and approved the submitted version.

## Conflict of Interest

The authors declare that the research was conducted in the absence of any commercial or financial relationships that could be construed as a potential conflict of interest.

## Footnotes

^1^In the original scale these were items 1, 5, 6, and 8. Running the analyses for the present study with the original powerlessness scale and all items included yielded similar results and led to identical conclusions.
